# Outcome of pelvic ring injuries

**DOI:** 10.1007/s00402-024-05606-w

**Published:** 2024-12-16

**Authors:** Axel Gänsslen, Jan Lindahl, Dietmar Krappinger, Richard A. Lindtner, Mario Staresinic

**Affiliations:** 1 Department of Trauma Surgery, Hannover Medical School, Hanover, Germany; 2 Johannes Wesling Klinikum Minden, Minden, Germany; 3 Helsinki University Hospital, Helsinki, Finland; 4 Innsbruck Medical University, Innsbruck, Austria; 5 University Hospital Merkur, Zagreb, Croatia

**Keywords:** Pelvic ring injury, Treatment concept, Outcome evaluation, Stabilization methods

## Abstract

Outcome evaluation is of major importance to provide data to analyze the value of the chosen treatment concept. Despite an increasing effort of analyzing outcome after treatment of different pelvic ring injuries, a mixture of different outcome parameters is in use. The Majeed score is most frequently used for mid- to long-term evaluation and the quality of life is analyzed using the SF-36 score. The lack in nearly all studies is that different treatment concepts are used, and only selected evaluation parameters are reported. Until today, no well-accepted standardized measurement instruments are available to analyze the clinical and radiological results after pelvic ring injuries. Overall, stability-based long-term sequelae can be expected with increasing complaints from stable type A injuries to completely unstable type C injuries. Beside a fracture-type specific treatment, concomitant injuries of other injury regions and associated local pelvic injuries (complex pelvic trauma) seem to additionally influence the results. Results of treatment of specific fracture types are sparse as a wide range of different injury types and different treatment concepts are analyzed within these analyses. A sufficient pelvic outcome instrument which addresses relevant pelvic outcome parameters is still missing. Thus, future evaluation of long-term results after pelvic ring. injuries should include prospective, multicenter outcome studies with comparable parameters.

## Introduction

Adequate long-term follow-up analyses of results of treatment of pelvic ring fractures are missing in the scientific literature as the majority of reports are only dealing with emergency management and initial treatment, describing several techniques to treat these injuries and reporting on short-term results including surgery-related complications and quality of reduction.

Relevant parameters that may influence the long-term outcome are usually not considered.

For example, an analysis of results of symphyseal plating can be influenced by several parameters, including fracture type, additional posterior stabilization, type of posterior stabilization, type of plate, local soft-tissue injuries, accompanying intrapelvic organ injuries, concomitant whole body injuries etc.

It is generally accepted to distinguish pelvic ring injuries as stable, partially stable, or unstable according to Marvin Tile’s classification or the AO/OTA classification. Therefore, basic analyses should be focused on fracture groups within this classification or even depending on a chosen stabilization method. Thus, several unselected outcome analyses were published in the literature since decades.

Additionally, evaluation of long-term results after bony injuries have focused on radiologic outcomes or nonvalidated measures of pain and function that did not facilitate comparison of results from one report to the next [44]. With the advent of validated measurement instruments, deficits in physical, social, and emotional functions subsequent to injury and disease now can be consistently quantified [45]. Several nonvalidated and validated outcome measures are used in describing the long-term results after pelvic ring fractures.

One major concern while analyzing the outcome after pelvic ring injuries is to evaluate at which time follow-up studies should be performed. Based on few analyses, follow-up studies for pelvic ring injuries should have at least a minimal follow-up evaluation of 2 years [9, 44, 57].

## Outcome intsruments

Different outcome instruments are in use for long-term evaluation of pelvic ring injuries.

Generic outcome instruments include: SF-36 [91], Short Musculoskeletal Functional Assessment (SMFA) [83], EuroQol Quality of Life Score (EQ-5) [11], Sickness Impact Profile (SIP) [39], Functional Independence Measure (FIM) [24], Life Satisfaction Scale (Lis-AT 11) [26], and the Hamilton Depression Rating scale (HDARS) [94].

Disease (pelvic)-specific instruments include: Majeed Score [56], Iowa Pelvis Score (IPS) [84], Hannover Pelvic Score (POS) [70–72], Orlando Pelvic Score [16], Pelvic Trauma Questionnaire (PTQ) [7–9] and Merle d’Aubigné Score [18].

## Unselected treatment results

It is well known, that there is a decrease of good and excellent results with increasing.

pelvic ring instability from type A to type C injuries [22, 72]. Neurological long-term sequelae increased with the type of pelvic ring injury with 4% after type A, 11% after type B, and 17% after type C injuries. Urological and sexual sequelae were both present in 8%, with highest rates after type C injuries (each 14%). Additionally, patients with less than 5 mm residual displacement showed best functional results with a Majeed Score of > 90, whereas with increasing displacement significantly lower scores were observed [22].

Pavelka et al. found comparable results in their single-center analysis. Excellent and good clinical outcomes according to the Majeed Score were seen in 83% after type B and in 70% after type C fractures. The radiological result was excellent (< 5 mm residual displacement) in 83% and 61%, respectively [66].

Other authors confirmed these results.

Type C injuries are associated with more clinical long-term discomfort [5, 6, 13, 27, 34, 36, 41, 48].

Based on a detailed literature research several more detailed analyses came to the following results for unselected analyses (different types of type C injuries, no distinction between classification, fracture types, fracture location, or treatment modalities) (overview in: [27, 30, 31]):

- *type B-injuries*: only minor impairments were observed compared to the normal population using a validated outcome instrument (SF- 36) [47, 48, 59, 64, 89]; despite a high radiological healing rate, approximately 30% long-term problems (pain, functional limitations) [58, 59, 72, 86], and an overall rate of 75–80% acceptable results can be expected [27].
- *type B1 injuries*: moderate disability was observed in 30–40% with relevant persistent pain, despite a high rate of anatomical healing; functional excellent and good results can be expected in 70–90% when symphyseal plating was performed, while external fixation seems to lead to worse results [32, 53, 69, 74, 89, 92].
- *type B2 injuries*: lateral compression injuries led to a low rate of permanent pain (5–15%), while anatomical healing of the pelvis was reported in only 70–75%; the overall functional outcome was rated excellent or good in 75–90% [35, 47, 53].
- *type C-injuries*: using a validated outcome instrument (SF-36), relevant impairments were observed compared to the normal population; operatively treated sacrum fractures showed better results than after pure SI joint dislocations [14, 48, 53, 59]; approximately 30–50% report significant persistent pain and have sexual or urogenital disturbances at follow-up leading to an excellent or good overall functional result can in 70–80%; healing with residual displacement < 5 mm was observed in 65–90%, single external fixation is considered an inadequate treatment modality [16, 48, 51, 53, 58, 60, 74, 85].

Compared to type B injuries, a nearly twofold higher rate of impairments can be expected [14, 59, 64]. The concept of anterior and posterior stabilization is recommended in type C injuries [16, 48, 51, 53, 58, 60, 74, 85].

These analyses are unselected and usually integrate different stabilization techniques even within a single analysis, ignore the effect of concomitant injuries and local fracture-related injuries. Additionally different outcome instruments were used.

Thus, an additional interest should focus on stabilization-specific and fracture-type specific outcome analyses.

## Stabilization-type specific outcomes

A focus in the literature was observed for specific posterior stabilization methods, e.g. iliosacral screw fixation, anterior SI-joint plating, ilioiliacal stabilization techniques and lumbopelvic fixation techniques. In summary, the following results were reported, but only few analyses dealed with such results:

- *iliosacral screw fixation*: studies predominantly focus on quality of reduction, safety of screw positioning, radiation exposure, and perioperative parameters [12, 100], while the long-term result after iliosacral screw fixation of posterior pelvic instabilities remains unclear [42, 78, 98].
- *anterior SI-joint plating*: while anatomical healing is observed in 80%, approximately half of these patients present with relevant functional limitations [29, 73].
- *ilioiliacal stabilization techniques*: percutaneous techniques led to 70–75% good to excellent clinical results with near anatomical healing (< 5 mm) was observed in 50–80% [3, 21, 25, 43, 82].
- *lumbopelvic fixation techniques*: good to excellent clinical results were reported in only 65% with low rates of loss of reduction (85–87); in spinopelvic fracture dislocations excellent and good results were reported in < 70% possibly due to the permanent nerve injury and a high rate of persistent pain [4, 19, 49, 50, 76].

## Posterior fracture-type specific outcomes

Additionally, few outcome data were identified for specific posterior fracture types, e.g. complete iliac fractures (type C1.1.), pure SI-joint dislocations (type C1.2) and unstable sacral fractures (type C1.3):

- complete ilium fractures: no clear outcome data are available [14, 16].
- pure SI-joint dislocations: 30–40% persistent pain is reported and half of these patients have relevant functional impairments [73, 85].
- sacral fractures: > 50% of patients report some pain with an overall rate of excellent or good functional result in 65–75% [3, 63, 82, 85].

## Prognostic factors influencing long-term result

Several prognostic factors of worse clinical and functional outcomes after pelvic injuries are well described in the literature and include residual displacement, persistent nerve injury and additional pelvic soft-tissue injury (complex pelvic trauma [10]). Based on the reported data until 2017 the following results can be derived from the literature:

- the primary treatment aim in type C-injuries is a residual posterior displacement of ≤ 5 mm [1, 17, 62, 72, 85].
- unstable sacral fractures or spinopelvic dissociation injuries result in relevant rates of persistent nerve deficits [15, 16, 21, 27, 43, 52, 71, 72, 78, 81].
- concomitant pelvic soft-tissues injuries including pelvic organ injuries influence the clinical and functional long-term outcome [14, 17, 23, 25, 28, 33, 70–72, 77].

## New analyses

In the last recent years, several studies dealed with specific outcome questions. Details of these studies are presented and potentially discussable topics are analyzed (shown in italic). Additionally, some systematic analyses were performed on specific treatment questions.

### Systematic reviews

In a systematic review on managing unstable anterior pelvic ring injuries with the subcutaneous screw rod system (INFIX) 619 patients from 22 studies were analyzed [46]. 87% excellent to good radiological results and 84% excellent to good functional results were reported. Relatively high complication rates were reported: 25.3% lateral femoral cutaneous nerve irritation, 24.7% local heterotopic ossification, 3% infection and 1.6% femoral nerve palsy.

*Different fracture types were included in this analysis (Tile type B and C). Pelvis-specific outcome instruments included the Majeed score in the majority of reports*,* but also the IOWA score and the Hannover outcome instrument was used. No data on posterior ring stabilization was distinguished (e.g. performed posterior stabilization*,* type of stabilization*,* approach (open vs. minimal-invasive). Thus*,* the statement*,* that “INFIX is a reliable technique for anterior pelvic injury” does not take into account potential relevant parameters. Radiographic parameters and outcome measures were infrequently reported.*

One year later, an additional systematic review was published, comparing the INFIX technique vs. anterior plating in unstable pelvic ring injuries after high‑energy trauma in at least 10 patients [65]. Follow-up ranged from 5 to 54 months. INFIX was associated with 28% lateral femoral cutaneous nerve irritation, 9.4% local heterotopic ossification. Excellent and good radiological results were observed in 91.4%, and the functional outcome was good with a mean Majeed score of 86.48.

*Fracture types included Tile type B and C injuries without distinguishing results to specific fracture types. No information was presented in terms of posterior stabilization methods and posterior vs. anterior radiographic results. Thus*,* the clinical outcome remains unclear.*

In an analysis of unstable LC-1 injuries with complete sacral fractures, 183 surgically treated patients were compared with 314 patients treated non-operatively [90]. After operative stabilization less pain and fewer days to mobilization were observed with a better non-significant quality of life result (EuroQol-5, SF-36). *No clear stabilization concept could be identified from these data (isolated anterior*,* combined anterior/posterior or isolated posterior and it remained unclear why several patients were classified as Tile type B injuries in patients with a defined unstable sacral fracture (formally Tile type C). The grey zone between the Young-Burgess classification and the Tile classification potentially influenced the results of this analysis.*

In a recent systematic review on APC type II injuries different indication for combined anterior/posterior or isolated anterior fixation were reported [38]. It was stated, that “No reliable evidence was found to support either SP (anterior plate fixation) or SP + SIS (additional posterior sacroiliac screw) as the treatment of choice. The reviewed clinical data suggest that SP + SIS could reduce implant failure, revision surgery rates and could improve fixation success in APC II pelvic ring injuries”.

Comparing triangular osteosynthesis and classical lumbopelvic fixation, a meta-analysis on 212 patients with sacral fractures reported an effectiveness of both stabilization methods [75]. Only Tile type C injuries were part of this analysis but additionally several lumbopelvic dissociation injuries were integrated. *No detailed analysis of outcome was reported*,* despite concluding an adequate quality of life postoperatively.*

Despite interesting systematic reviews were performed to answer specific questions comparing treatment method, all studies, analyzed different fracture types and stabilization methods at either the anterior or posterior ring.

### Results after tile type C-injuries

Liu et al. compared percutaneous anterior INFIX vs. plating for Tile type C injuries with all patients having posterior iliosacral screw fixation [54]. At > 12 months follow-up the resulting clinical (Majeed score) and radiographic results (Matta scoring) was graded excellent and good in 100% in both groups. The minimal invasive concept resulted in a reduced operative time, intraoperative blood loss and transfusion, incision length and hospital stay. *Different anterior approaches were used (lateral rectus abdominis approach*,* intrapelvic approach).*

Deng compared single or double iliosacral screw fixation in Tile type C1 injuries assisted by a percutaneous robotic navigation system [20]. Follow-up was performed after 1an average of 6–17 months. Two iliosacral screw provide a more stable treatment without any effect on functional results. *For anterior pelvic ring fixation*,* different fixation concepts were used but not all patients were stabilized. Additionally*,* all fracture subgroups (Tile type C1.1.*,* C1.2 and C1.3) were included in this study.*

Unselected analyses are still performed in Tile type C injuries with integration of different fracture types and different fixation concepts.

## Fracture-type specific results

Several papers focused on fracture-type specific results. Especially lateral compression fracture types were of interest.

*LC-1 fractures (unstable lateral compression injuries*,* Tile type B and C)*: based on hospital outcome and short-term follow-up displacement, combined anterior and posterior stabilization was recommended [87]. *No clear differentiation is possible in terms of Tile type B and C injuries and type of stabilization*.

*LC-1 fractures (minimally displaced)*: non-operative treatment of comminuted anterior ring fractures was associated with secondary displacement ≥ 5 mm in an analysis of 125 patients [88]. *This study includes complete sacral fractures and comminuted sacral fractures and the indication for operative/non-operative treatment were not reported in detail.*

*LC-2 fractures (posterior transiliac fractures dislocations*,* Tile type C-injuries)*: isolated posterior fixation led to comparable good and excellent functional outcomes (Majeed score) compared to combined anterior and posterior stabilization. After 13–14 months, no difference between time to weight bear and time to clinical healing was reported, but clinically irrelevant anterior fracture displacement of up to 3 mm was reported after isolated posterior fixation [99].

*B2.1 injuries (lateral compression injuries)*: Petryla et al. compared operative and non-operative treatment in young patients (average age 37.24 years) [68]. Operative treatment differed: 23x combined anterior and posterior stabilization, 13x posterior stabilization only and 1x anterior external fixation. Operative treatment resulted not in better functional (Majeed score) and quality of life (SF-36). *Indications for surgical treatment remain unclear and the follow-up interval was only 10 weeks.*

An additional analysis after 1 year showed no differences in terms of quality of life and functional outcomes comparing isolated posterior pelvic ring fixation and combined anterior-posterior fixation [67].

*crescent fractures (Tile type C1.1)*: 64 patients with crescent fracture dislocation a subtype of LC fractures) were compared regarding the chosen approach (anterior vs. posterior) [95]. At a mean follow-up of 30 months the average Majeed score was > 87 points in both groups. Fracture dislocations grade Day III showed overall good results but were slightly worse than Day I and Day II fracture dislocation. The posterior approach achieved a better sacroiliac joint reduction especially in Day I fractures, while in Day III fractures the overall result was approach-independent. *All fractures were graded as Tile type B injuries (?) as usually the posterior pelvic ring is completely unstable. Different anterior and posterior stabilization methods were chosen*.

*LC-fractures*: in an analysis of LC fractures in 25 young patients (mean age 29.8 years) including all three types of LC fractures, all patients were stabilized only posterior [2]. The median Majeed score was 90 and no difference in terms of quality of life (SF-36) was reported. 80% of good and excellent functional outcomes could be expected. *No fracture-type specific analysis was performed and the morphology of the anterior ring injury was not reported.*

*unstable sacral Tile type C fracture*: in 19 obese patients posterior pelvic ring fixation was performed with several fixation constructs leading to 73.7% excellent and good posterior reductions [55]. Follow-up at an average of 41 months showed an average Majeed score of only 66.5 and an SF-12 score of 74.32. *No information was presented regarding anterior ring injury and treatment. Six different posterior fixations methods were used.*

There is still a mix of different fracture types and no universal treatment concept is present, leading to problems in answering of specific questions in terms of outcome.

Analysis of the presented studies showed, that the group of LC-1 fractures is insufficiently defined [87, 88]. Several studies distinguish between unstable and stable fracture types leading to inconsistent recommendations. These fractures types belong to the fracture groups B and C according to the Tile and AO/OTA classification.

## Combined anterior and posterior stabilization

Based on the literature, combined anterior and posterior fixation is commonly recommended in type C injuries.

In LC-2 fractures (posterior transiliac fractures dislocations, Tile type C-injuries) isolated posterior fixation led to comparable good and excellent functional outcomes (Majeed score) similar to combined anterior and posterior stabilization [99]. *Not surprising*,* isolated posterior fixation was associated with less intra-operative blood loss*,* less intra-operative fluid administration*,* less postoperative drainage*,* a reduced rate of blood transfusion and two days less hospital stay. Follow-up was performed in average after 13–14 months. No difference between time to weight bear and time to clinical healing was reported. Isolated posterior fixation was associated with anterior fracture displacement of up to 3 mm*.

Liu et al. compared a percutaneous approach for Tile type C injuries with anterior INFIX vs. plating and posterior iliosacral screw fixation [54]. At < 12 months follow-up the resulting clinical (Majeed score) and radiographic results (Matta scoring) was graded excellent and good in 100% in both groups. The minimal invasive concept resulted in a reduced operative time, intraoperative blood loss and transfusion, incision length and hospital stay. *Different anterior approaches were used (lateral rectus abdominis approach*,* intrapelvic approach).*

In an analysis of anterior straddle fracture combined with posterior pelvic ring injury not all patients were treated with posterior stabilization methods [96]. *Different posterior stabilization methods were used*,* anterior stabilization was not uniformly performed and this analysis included Tile type B and C injuries. Posterior displacement was differentiated to be > 1 cm or < 1 cm*,* without a clear trend in terms of indication of posterior stabilization. A detailed outcome analysis (follow-up > 1 year*,* Majeed score) was reported for specific fracture combinations*,* but without distinction of the fracture type.*

In a further analysis, this group compared unilateral vs. bilateral ramus fixation in 118 patients with posterior pelvic ring injury treated operatively [97]. Bilateral fixation was not associated with better short-term results. *Again*,* this analysis included Tile type B and C injuries*,* different posterior stabilization methods without adequate long-term results.*

An analysis after 1 year in Tile type B2 injuries showed no differences in terms of quality of life and functional outcomes comparing isolated posterior pelvic ring fixation and combined anterior-posterior fixation [67]. *Indications for type of surgical treatment remain unclear and no clear concept was reported in terms of stabilization method.*

Moussa et al. compared combined anterior and posterior ring fixation versus single posterior ring fixation in 46 Tile B2 and C1 pelvic ring injuries [61]. Isolated posterior fixation lead to comparable results to combined fixation but with less morbidity.

There is a growing interest whether to stabilize only the posterior pelvic ring in unstable pelvic ring injuries. Different stabilization concepts are reported and no distinction between fracture types was made.

## Posterior stabilization methods

In an analysis comparing posterior iliosacral screw fixation (ISF) and lumbopelvic fixation (LPF) in bilateral posterior sacral fractures and additional anterior ring fixation (uni-or bilateral superior ramus screw) LPF led to earlier full weight bearing but with later discharge time [93]. ISF was associated with more screw mal-position, hardware change and implant removal but led non-unions.

*Beside classical bilateral longitudinal fractures*,* 18% of patients had formally spinopelvic dissociation injuries. LPF fixation was performed in younger patients while ISF was performed in elderly/geriatric patients.*

### Iliosacral screw fixation

Deng compared single or double iliosacral screw fixation in Tile type C1 injuries (Tile type C1.1., C1.2 and C1.3) assisted by a percutaneous robotic navigation system [20]. Follow-up was performed after 1an average of 6–17 months. Two iliosacral screw provide a more stable treatment without any effect on functional results. *For anterior pelvic ring fixation*,* different fixation concepts were used but not all patients were stabilized. Additionally*,* all fracture subgroups (Tile type C1.1.*,* C1.2 and C1.3) were included in this study. Operation time was reported (robotic assistance) to be more than 3 h in average!*

Shaalan et al. analyzed the results of 25 patients with unstable sacral fractures stabilized with posterior iliosacral screw fixation [79]. After a mean of 14.8 months, the mean Majeed score was 82.16 points with 23 patients (92%) presenting with an excellent or good functional result. The radiographic results (Matta criteria) were graded excellent in 17 patients (68%) and good in five (20%).

In an analysis of 84 patients after high-energy trauma and iliosacral screw fixation 23 patients with type B2 fractures, seven with type B3 fractures 39 with type C1 fractures, eight with type C2 fractures and seven with type C3 fractures were included [80]. After a median follow-up of 130.1 months the mean Majeed Score was 83.32 points and difference between uni- and bilateral pelvic fractures was reported. *The definition of bilateral fractures was not clearly reported and no clear data were reported for anterior ring treatment.*

### Ilioiliacal fixation

Shaalan et al. analyzed the results of 25 patients with unstable sacral fractures stabilized with ilioiliacal internal fixation (TIFI technique) [79]. After a mean of 16.6 months, the mean Majeed score was 80.46 points with 23 patients (88.5%) presenting with an excellent or good functional result. The radiographic results (Matta criteria) were graded excellent in 15 patients (57.7%) and good in eight (30.8%). *The type and frequency of anterior ring stabilization was individualized.*

### triangular osteosynthesis

in a mid-term analysis (mean follow-up 3.1 years) of 22 patients with predominantly unilateral posterior pelvic ring injuries, 20 patients showed good to excellent functional results (Majeed score, IOWA score) [37]. In all but one patient the Majeed score was far above 80 points. An individual stabilization concept was used with two patient stabilized anteriorly.

Stabilization-specific results have still the lack of individual stabilization concepts (analysis of different fracture types and integration of different fixation concepts).

## Which residual displacement can be accepted

Despite data from the last decades propose posterior reconstruction in unstable pelvic ring injuries to be within a limit of 5 mm displacement, a recent analysis accepted up to 10 mm of residual displacement in pelvic ring injuries. This recommendation was based on analysis of 49 patients treated operatively and non-operatively with different fracture types according to the Young-Burgess classification (all fracture types except combined mechanism were reported) [40].

*No fracture-specific treatment concept was reported. Patients treated non-operatively were rated better using the Majeed score.*

## Conclusion

Despite an increasing effort of analyzing outcome after treatment of different pelvic ring injuries, no clear outcome data are available as different treatment concepts are used, and the majority of published studies are not comparable in terms of selected evaluation parameters.

No well-accepted standardized measurement instruments are available to analyze the clinical and radiological results after pelvic ring injuries. Only few analyses present adequate data.

Overall, an increase of long-term sequelae can be expected from stable type A injuries to completely unstable type C injuries. Type B1 injuries lead to worse results than type B2 injuries. Beside the fracture-type specific treatment, concomitant injuries of other injury regions and associated local pelvic injuries (complex pelvic trauma) seem to additionally influence the results. Results of treatment of specific fracture types are sparse as a wide range of different injury types and different treatment concepts are analyzed.

A sufficient pelvic outcome instrument which addresses relevant pelvic outcome parameters is still missing. Thus, future evaluation of long-term results after pelvic ring.

injuries should include prospective, multicenter outcome studies.

## Data Availability

No datasets were generated or analysed during the current study.
